# PAX2 Expression in Ovarian Cancer

**DOI:** 10.3390/ijms14036090

**Published:** 2013-03-15

**Authors:** Huijuan Song, Suet-Yan Kwan, Daisy I. Izaguirre, Zhifei Zu, Yvonne T. Tsang, Celestine S. Tung, Erin R. King, Samuel C. Mok, David M. Gershenson, Kwong-Kwok Wong

**Affiliations:** 1 Department of Gynecologic Oncology and Reproductive Medicine, The University of Texas MD Anderson Cancer Center, Houston, TX 77030, USA; E-Mails: shj80929@gmail.com (H.S.); skwan@mdanderson.org (S.-Y.K.); dizaguir@mdanderson.org (D.I.I.); zzu@mdanderson.org (Z.Z.); ytsangle@mdanderson.org (Y.T.T.); ctung@bcm.edu (C.S.T.); erking@mdanderson.org (E.R.K.); scmok@mdanderson.org (S.C.M.); dgershen@mdanderson.org (D.M.G.); 2 Cancer Biology Program, The University of Texas at Houston Graduate School of Biomedical Sciences, Houston, TX 77030, USA

**Keywords:** PAX2, ovarian cancer, G0S2, WFDC1, GREM1, shRNA

## Abstract

*PAX2* is one of nine *PAX* genes that regulate tissue development and cellular differentiation in embryos. However, the functional role of PAX2 in ovarian cancer is not known. Twenty-six ovarian cancer cell lines with different histology origins were screened for PAX2 expression. Two ovarian cancer cell lines: RMUGL (mucinous) and TOV21G (clear cell), with high PAX2 expression were chosen for further study. Knockdown PAX2 expression in these cell lines was achieved by lentiviral shRNAs targeting the *PAX2* gene. PAX2 stable knockdown cells were characterized for cell proliferation, migration, apoptosis, protein profiles, and gene expression profiles. The result indicated that these stable PAX2 knockdown cells had reduced cell proliferation and migration. Microarray analysis indicated that several genes involved in growth inhibition and motility, such as G0S2, GREM1, and WFDC1, were up-regulated in PAX2 knockdown cells. On the other hand, over-expressing PAX2 in PAX2-negative ovarian cell lines suppressed their cell proliferation. In summary, PAX2 could have both oncogenic and tumor suppression functions, which might depend on the genetic content of the ovarian cancer cells. Further investigation of PAX2 in tumor suppression and mortality is warranted.

## 1. Introduction

*PAX2* is one of nine *PAX* genes; all of these genes have a conserved DNA sequence motif called the paired box that comprises a 128-amino acid domain in the amino-terminal portion of the protein [1]. During embryogenesis, *PAX2* is abundantly expressed in the kidney [2,3], ureter [4], eye [5], cochlea [6], pancreas [7], and central nervous system [8,9] and is crucial to embryogenic development, morphogenesis, and organogenesis [10,11]. Embryonic *PAX2* gene expression is largely attenuated in adult tissue although continued expression can be detected in female genital tract, breast and other tissues [12]. *PAX2* deficiency has been associated with various growth defects, such as kidney hypoplasia, optic coloboma, and vesicoureteral reflux [13]. Conversely, PAX2 overexpression is associated with cystic or tumorous epithelial overgrowth [14], such as renal cystic dysplasia, renal cell carcinoma, Wilms’ tumor, nephrogenic adenoma, prostate cancer, breast cancer and ovarian cancer [15–19]. Expression of PAX2 in these cells appears to be important for tumor cell survival [17,20,21]. However, recent studies indicated that loss of PAX2 expression correlates with the development of serous carcinoma in the fallopian tube [22–24]. Similarly, the loss of PAX2 expression also correlates with the development of endometrial precancer and cancer [25]. Thus, it is possible that PAX2 could be an oncogene or tumor suppressor [12]. The function of *PAX2* in the development of ovarian cancer is still unknown. In this study, using both PAX2 positive and negative ovarian cancer cell lines, we investigated the potential functional roles of PAX2 in ovarian cancer.

## 2. Results

### 2.1. Ovarian Cancer Cell Lines Expressed Different Level of PAX2

**Figure 1:**
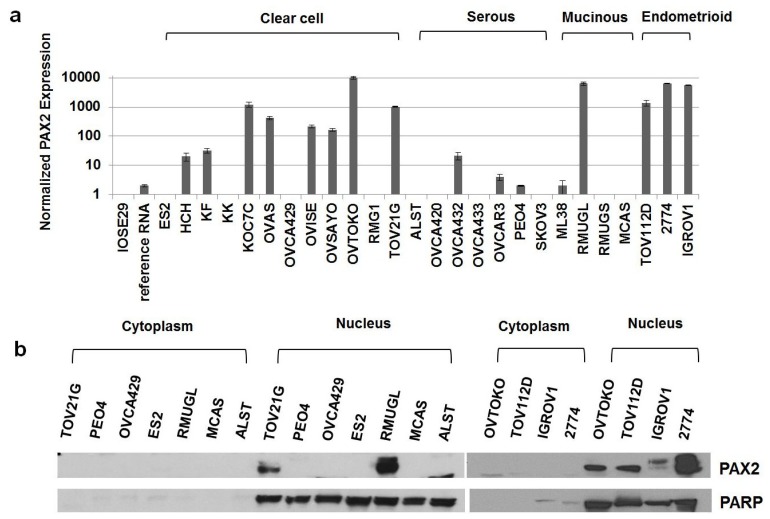
Expression of PAX2 in various ovarian cancer cell lines. (**a**) Real-time RT-PCR analysis of *PAX2* mRNA expression in twenty-six ovarian cancer cell lines with different histology origins; (**b**) Western blot analysis of PAX2 protein expression level in seven selected ovarian cancer cell lines.

Twenty-six ovarian cancer cell lines (8 serous ovarian cancer cell lines, 12 clear ovarian cancer cell lines, 3 mucinous ovarian cancer cell lines, 3 endometrioid ovarian cancer cell lines) and one immortalized normal ovarian surface epithelium cell line (IOSE29) were screened for *PAX2* expression by real-time RT-PCR. Sixteen of the cell lines (HCH, KF, KOC7C, OVAS, OVISE, OVSAYO, OVTOKO, TOV21G, OVCA 432, OVCAR3, PEO4, ML38, RMUGL, TOV112D, 2774, and IGROV1) exhibited 2 to 10270 times higher *PAX2* mRNA expression than IOSE29 cells did. *PAX2* was found to be highly expressed in mostly non-serous ovarian cancer cell lines (Figure 1A). RMUGL and OVTOKO had the highest expression level of *PAX2* mRNA followed by 2774, IGROV1, KOC7C, TOV112D and TOV21G. Figure 1B showed the nuclear protein expression of PAX2 expression by Western blot in a few selected ovarian cancer cell lines with different level of mRNA expression. There is a strong correlation of PAX2 protein expression with the mRNA expression.

### 2.2. PAX2 Knockdown Is Associated with Reduced Cell Proliferation

**Figure 2:**
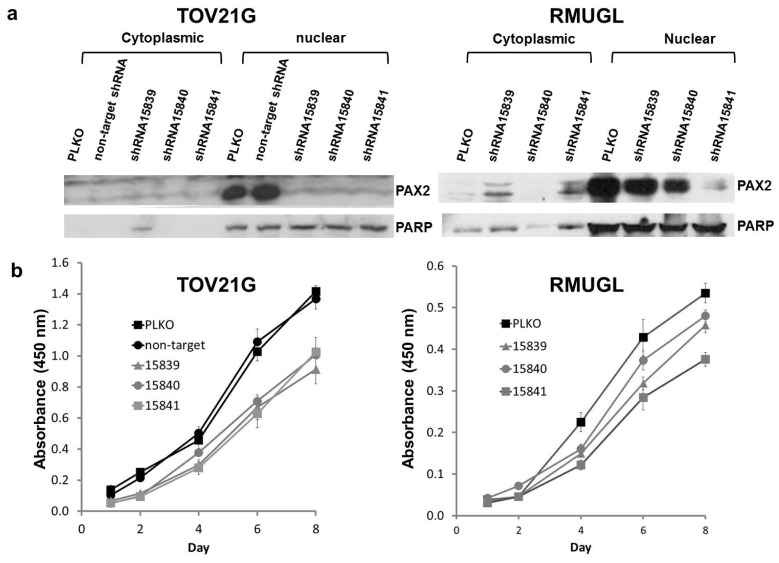
(**a**) Western blot analysis of TOV21G clear cell line and RMUGL mucinous cell line stably transfected with PAX2-targeted shRNAs 15839, 15840, and 15841. PLKO and non-target are vector controls; (**b**) Cell proliferation of PAX2 shRNAs stably transfected cells measured by WST-1 assay.

Two PAX2 positive cell lines of different histology origins (RMUGL and TOV21G) and of different levels of PAX2 expression were chosen for *PAX2* knockdown experiments. MISSION TRC shRNA lentiviral particles (three independent shRNAs—15839, 15840 and 15841) were used to transduce the ovarian cancer cell lines RMUGL and TOV21G. After selecting stably transfected cells by puromycin, Western blotting was used to evaluate the PAX2 knockdown efficiency (Figure 2). For RMUGL cell line, PAX2 expression was partially knockdown in shRNA 15839- and shRNA 15840-stably transfected cells, but almost completely knockdown in shRNA 15841-stably transfected cell (Figure 2a). For TOV21G cell line, PAX2 expression was completely knockdown in all PAX2 shRNA stably transfected cells (Figure 2a). The knockdown efficiency was especially robust using shRNA 15841. The difference in PAX2 knockdown efficiency is likely due to a 10-fold higher expression of *PAX2* in parental RMUGL cell line than TOV21G cell line.

Cell proliferation assays were performed on both the original cell lines and the PAX2 stably knockdown cell lines. As shown in Figure 2b, the cell proliferation rate of the most PAX2 knockdown RMUGL cell line (shRNA 15841) had a significant decrease in cell proliferation rate in comparison to the PLKO vector-transfected control cells while the decrease in cell proliferation in other two shRNAs (15839 and 15840) stably transfected RMUGL was not significant. This suggested a near complete knockdown of PAX2 might be necessary to affect cell proliferation. The effect of PAX2 knockdown on cell proliferation was significant (*p* < 0.05) in the clear cell ovarian cancer cell line TOV21G (Figure 2b).

### 2.3. PAX2 Knockdown Is Associated with Reduced Cell Motility

**Figure 3:**
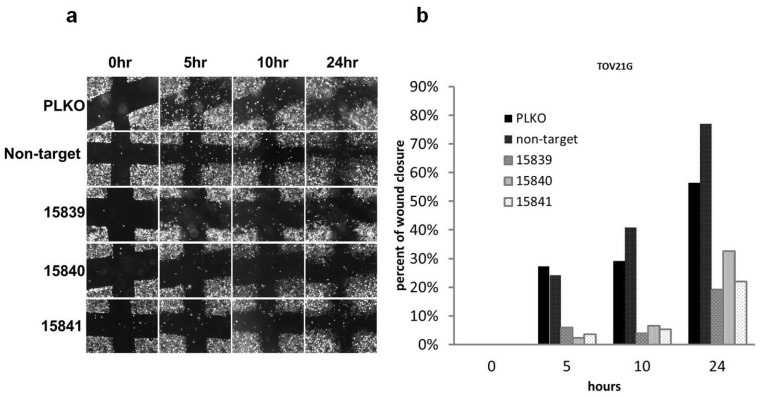
(**a**) Images of wound closure assay of TOV21G cell line stably transfected with PAX2 specific shRNAs during a 24 h period. PLKO and non-target shRNA were the negative controls, and shRNAs 15839, 15840 and 15841 were PAX2-targeted shRNAs; (**b**) the images were analyzed by the TScratch program [26] to estimate the percentage of wound closure. PAX2 stable knockdown TOV21G cells had slower rate of wound closure.

To test whether PAX2 plays a role in cell migration, we conducted a wound healing assay using the TOV21G cell lines. Compared with control cell lines with positive PAX2 expression, PAX2 knockdown cell lines had reduced cell motility (Figure 3). Similar results were obtained with PAX2 knockdown RMUGL cells by shRNA 15841 (data not shown).

### 2.4. Cell Signaling Protein Expression Analysis by Reverse Phase Protein Array (RPPA) Suggested PAX2 Knockdown Might Enhance Apoptotic Signaling

**Figure 4:**
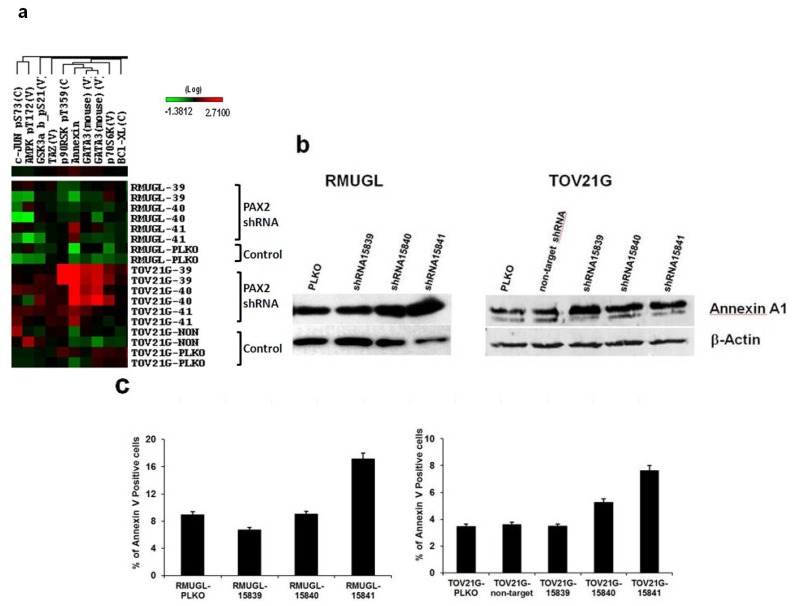
Up-regulation of Annexin A1 and increase in apoptotic cells in PAX2 knockdown cell lines. (**a**) Reverse phase protein array (RPPA) analysis showed PAX2 knockdown ovarian cancer cell lines had a higher expression of Annexin A1 than control cells; (**b**) Western blot analysis was used to measure Annexin A1 expression in RMUGL and TOV21G ovarian cancer cell lines with or without PAX2 knockdown; (**c**) Flow cytometric analysis of percentage of apoptotic cells using APC-Annexin V staining.

RPPA was used to analyze the effects of PAX2 knockdown on the expression of 207 signaling proteins in ovarian cancer cell lines RMUGL and TOV21G. Two independent protein lysates replicate were analyzed. RPPA sample preparation, slide printing, staining and data analysis were processed as described [27]. From the RPPA analysis, PAX2 knockdown cell lines had increased expression of Annexin A1, a marker of early stage apoptosis (Figure 4a). Annexin A1 expression was further confirmed by Western blot (Figure 4b). To evaluate the potential role of PAX2 in apoptosis, allophycocyanin-Annexin V staining, which detects an increase of phosphatidylserine residues in the outer plasma membrane leaflet during the early stages of apoptosis was used to measure apoptotic cells in RMUGL and TOV21G ovarian cancer cell lines with PAX2 knockdown (Figure 4c). In RMUGL, the percentage of apoptotic cells was 6.73%, 9.01%, and 17.15% for cells transfected with shRNAs 15839, 15841, and 15841, respectively, compared with 8.95% in the PLKO control (Figure 4c). In TOV21G, the percentage of apoptotic cells was 3.48%, 5.27% and 7.63% for cells transfected with shRNAs 15839, 15841, and 15841, respectively, compared with 3.47% in the PLKO control (Figure 4c). Thus, there was an association between the extent of PAX2 knockdown and the percentage of apoptotic cells.

### 2.5. *G0S2*, *WFDC1* and *GREM1* Were Up-Regulated in PAX2 Knockdown TOV21G Cell Lines

**Figure 5:**
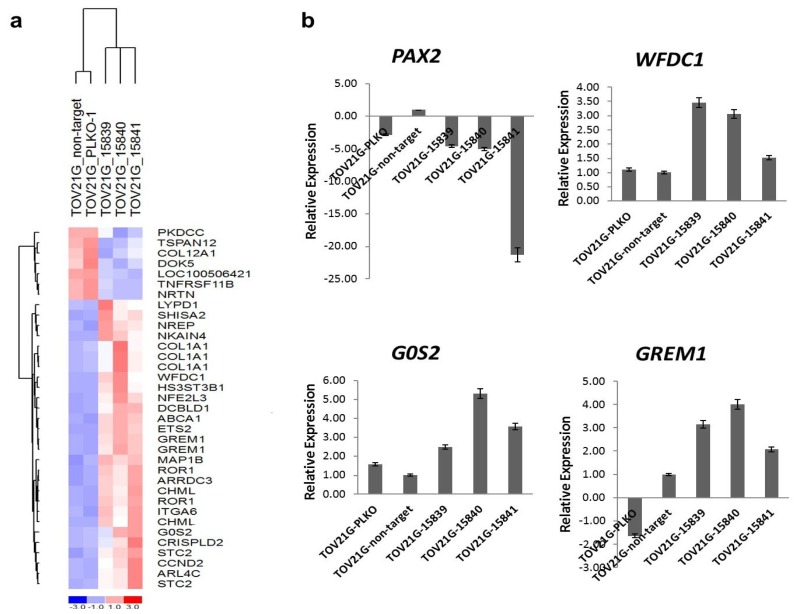
(**a**) The most differentially expressed genes in the PAX2 stable knockdown TOV21G clear cell ovarian cancer cell lines identified by microarray analysis; (**b**) Validation of down-regulation of *PAX2* and the up-regulation of *G0S2*, *WFDC1*, and *GREM1* in PAX2 knockdown TOV21G cell lines by RT-PCR.

Potential genes affected by PAX2 knockdown were identified by gene expression profiling of TOV21G cells with PAX2 knockdown by shRNA 15839, 15840, and 15841; these expression profiles were compared to that of TOV21G-PLKO amd TOV21G-non-target control cells. Gene expression data has been deposited in the public database (Gene Expression Omnibus, GSE30501). Genes that are highly differentially expressed between PAX2 knockdown TOV21G cells and control cells were shown (Figure 5a). The up-regulation of three genes involved in suppressing cell proliferation and/or cellular movement (*G0S2*, *WFDC1* and *GREM1*) was further validated by real-time RT-PCR (Figure 5b). The top 100 differentially expressed genes were listed in the Table S1.

### 2.6. Over-Expressing PAX2 Protein in PAX2-Negative Ovarian Cancer Cell Lines Is Associated with Reduced Cell Proliferation

**Figure 6:**
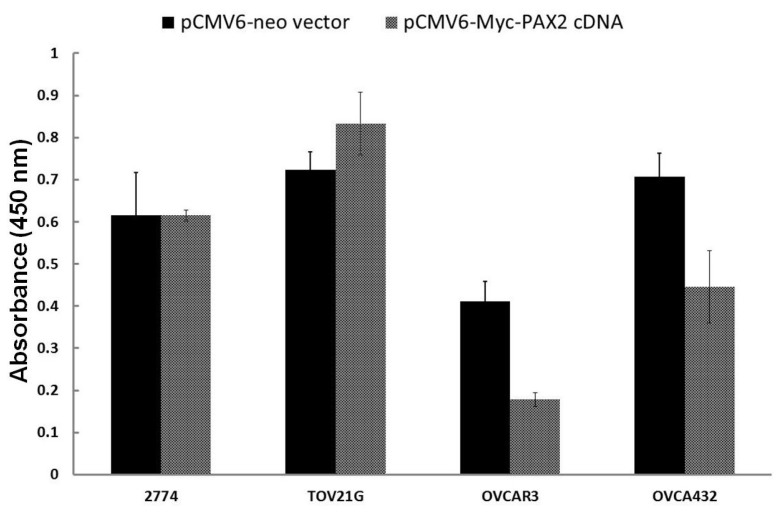
Cell viability of pCMV6-Myc-PAX2 transfected ovarian cancer cells. 2774 and TOV21G are PAX2-positive cells, while OVCA432 and OVCAR3 are PAX2-negative cells. The reduction in cell viability of OVCAR3 and OVCA432 cells after transfected with pCMV6-Myc-PAX2 plasmid in comparison to pCMV6-Neo vector control was significant (*p* < 0.05).

Two PAX2 negative serous ovarian cancer cell lines (OVCAR3 and OVCA432) after transfected with pCMV6-Myc-PAX2, cell proliferation was significantly suppressed in comparison to the vector transfection control (Figure 6). The cell numbers of pCMV6-Myc-PAX2 transfected OVCA432 cells were only 43% of pCMV6-Neo Vector transfected OVCA432. Similarly, pCMV6-Myc-PAX2 transfected OVCAR3 cells were only 63% of pCMV6-Neo vector transfected OVCAR3. On the other hand, there was no growth inhibition for two PAX2-positive cell lines (2774 and TOV21G) transfected by pCMV6-Myc-PAX2 plasmid DNA (Figure 6). Transfection experiments were repeated at least three times. Over-expression of PAX2 protein in the nuclear extract from transfected 2774 and TOV21G cells by pCMV6-Myc-PAX2 were confirmed by Western blot (data not shown).

### 2.7. Analysis of PAX2 Protein Expression in Non-Serous Ovarian Cancer Patient Samples by Immunohistochemistry

**Figure 7:**
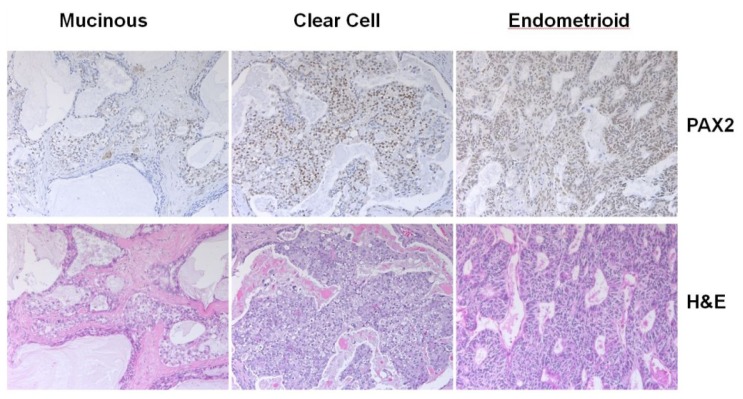
Examples of nuclear immunostains for PAX2 in a clear cell, mucinous and endometrioid ovarian carcinomas.

Immunostaining of PAX2 was performed on paraffin sections from 55 non-serous ovarian carcinomas. Nine out of 26 clear cell carcinomas were stained positive (9/26 = 35%), four out of 12 endometrioid carinomas were stained positive (4/12 = 33%), and two out of 17 mucinous carcinomas were stained positive (2/17 = 12%). Examples of positive nuclear staining were shown (Figure 7). For the clear carcinomas with PAX2 nuclear positive staining, the percentage of tumor cells with positive staining varied from 10% to over 75%. On the other hand, endometrioid carcinomas with PAX2 nuclear positive staining had percentage of tumor cells with positive staining from 50% to more than 75%. For the two mucinous carcinomas with positive staining, one had 5% tumor cells with positive staining, and the other one had more than 50% tumor cells with positive staining. Thus, the expression of PAX2 in clear cell and endometrioid carcinomas were significantly higher than that of serous carcinomas (9%) that we had reported previously [19]. The gene expression level of *PAX2* was estimated from microarray data (Figure S1), which indicated that non-serous ovarian carcinomas also had a higher gene expression of *PAX2*.

## 3. Discussion

In this study, we found that PAX2 is frequently expressed in ovarian cancer cell lines, especially for those derived from non-serous type ovarian cancers (clear cell, mucinous, and endometrioid). This is consistent with the *PAX2* expression in non-serous ovarian cancer from the gene expression data of ovarian cancer patient samples with different histological subtypes (GSE6008) and our own microarray data (Figure S1). This may reflect the different molecular pathogenesis or cellular origin of serous and non-serous ovarian carcinomas [28]. We have previously shown that PAX2 did not express in normal ovarian surface epithelia but expressed in ciliated epithelial inclusion in the ovaries and epithelia cells of the fallopian tube [19]. It is possible that some non-serous carcinomas are derived from ciliated epithelial inclusions. Ovarian clear cell and endometrioid carcinomas frequently carry *ARID1A* mutations but none of the high grade ovarian serous carcinomas have mutation in *ARID1A* [29]. On the other hand, almost all high grade ovarian serous carcinomas have mutated p53 but most ovarian clear cell and endometrioid carcinomas have wild-type p53 [28]. Recent study has shown that *PAX2* promoter activity is stimulated by wild-type p53 but inhibited by a dominant negative mutant p53 [30]. The binding of p53 to the chromatin regions of the *PAX2* promoter was identified by ChIP-Seq using developing kidneys in mice [30]. Thus, it is possible that the prevalent expression of PAX2 in non-serous carcinomas could be partly due to its activation by wild-type p53. However, further investigation will be necessary.

To determine the functional role of PAX2, knockdown PAX2 expression in PAX2-expressing ovarian cancer cells is associated with reduced cell proliferation and cell motility. The potential oncogenic role of PAX2 in these ovarian cancer cells is consistent with previous findings in prostate cancer, colon cancer, breast cancer and renal cancer [17,18,31,32]. Wound healing assays showed that PAX2 knockdown ovarian cancer cell lines had decreased cell motility in comparison to the parental PAX2-expressing cell lines. The reduced cell motility is further supported by the identification of more than 40 differentially expressed genes involved in cell proliferation and cell movement between the control cell lines and PAX2 knockdown cell lines. Three highly up-regulated genes (*G0S2*, *WFDC1*, and *GREM1*) in PAX2 knockdown cells were further validated by RT-PCR. G0S2 is a small basic nuclear phosphoprotein, one of the G0/G1 switch (G0S) genes that are differentially expressed in lymphocytes during lectin-induced switch from the G0 to the G1 phase of the cell cycle [33]. The expression of G0S2 is required to commit cells to enter the G1 phase of the cell cycle [34]. G0S2 specifically interacts with Bcl-2 and promotes apoptosis through preventing the formation of protective Bcl-2/Bax heterodimers [35]. WFDC1 (whey acidic protein four-disulfide core domain 1), a secreted protease inhibitor, and has been found it to be down-regulated in various cancers including fibrosarcomas, lung, bladder, and brain tumors [36]. Overexpression of WFDC1 has been shown to inhibit the growth rate of the fibrosarcoma HT1080 cell line [37]. GREM1 (Gremlin 1) is a member of the BMP (bone morphogenic protein) antagonist family [38]. Expression of GREM1 can regulate cancer cell growth positively [39] or negatively [40]. Further investigation of the roles of these up-regulated genes in PAX2 knockdown cells will be necessary to clarify their potential tumor suppression function in PAX2-expressing ovarian cancer cells.

The effects of PAX2 knockdown on cell signaling protein expression were studied using RPPA analysis. Annexin A1 was found to be the most significant up-regulated molecule detected by RPPA in PAX2 knockdown cell lines. Annexin A1 is proapoptotic by binding to the p65 subunit of NF-κB and thus inhibiting the NF-κB signal transduction pathway [41]. The increase in the percentage of apoptic cells in PAX2 knockdown cell lines were further demonstrated by flow cytometry with Allophycocyanin-Annexin V staining. Thus, the increased expression of Annexin A1 supports the notion that PAX2 may promote tumor growth in TOV21G ovarian cancer cell line by inhibiting the early stages of apoptosis through down-regulation of Annexin A1.

On the other hand, we have also shown for the first time that over-expressing PAX2 in PAX2-negative ovarian cancer cell lines suppresses their cell proliferation. Thus, PAX2 could have both oncogenic and tumor suppression functions which will depend on the genetic content of the cancer cells. However, the molecular mechanism is still unknown and will need further investigation.

## 4. Experimental Section

### 4.1. Cell Culture

Twenty-six human ovarian carcinoma cell lines (ES-2, HCH, KF, KK, KOC7C, OVAS, OVCA429, OVISE, OVSAYO, OVTOKO, RMG1, TOV21G, ALST, OVCA420, OVCA432, OVCA433, OVCAR3, PEO4, SKOV-3, ML38, RMUGL, RMUGS, MCAS, TOV112D, 2774, and IGROV1) and one immortalized ovarian surface epithelium cell line (IOSE29) were obtained from The University of Texas MD Anderson Cancer Center (Houston, TX, USA). All cells were grown in Roswell Park Memorial Institute-1640 (RPMI-1640) with 10% fetal bovine serum and 1% penicillin/streptomycin in 5% CO_2_ at 37 °C.

### 4.2. Stable PAX2 Knockdown Cell Lines

Twenty-six human ovarian carcinoma cell lines were screened for PAX2 expression by Western blots. Two cell lines (TOV21G, RMUGL) with robust PAX2 expression were used for generating stably PAX2 knockdown cell lines with PAX2 targeted shRNAs. MISSION TRC shRNA Lentiviral Particles (Sigma-Aldrich, St. Louis, MO, USA) targeting various regions of *PAX2* (shRNA 15839, CCGGCGTCTCTTCCATCAACAGAATCTCGAGATTCTGTTGATGGAAGAGACGTTTTT; hRNA 15840, CCGGCCCAAAGTGGTGGACAAGATTCTCGAGAATCTTGTCCACCACTTTGGGTT TTT; shRNA 15841, CCGGGATGAAGTCAAGTCGAGTCTACTCGAGTAGACTCGACTTGACT TCATCTTTTT) were used to infect the ovarian cancer cell lines that expressed PAX2. The control lentivirus PLKO-puro (no insert sequence) (Sigma-Aldrich, St. Louis, MO, USA) and shRNA non-target control (insert sequence: CCGGCAACAAGATGAAGAGCACCAACTCGAGTTGG TGCTCTTCATCTTGTTGTTTTT) (Sigma-Aldrich, St. Louis, MO, USA) were used as negative controls. Puromycin was used to select stable *PAX2* knockdown cells. After stable knockdown, Western blot and real-time reverse transcription polymerase chain reaction (RT-PCR) were used to confirm the efficacy of *PAX2* knockdown.

### 4.3. Gene Expression Profiling

TOV21G PLKO vector control, TOV21G non-target control, and TOV21G PAX2 knockdowns were profiled using Affymetrix Human Genome U133 plus 2.0 GeneChips (Affymetrix, Santa Clara, CA, USA) as previously described [42]. Raw expression images (CEL files) were processed using dChip software [43], and analysis was performed as described previously [44].

### 4.4. WST-1 Assay

The cell proliferation reagent WST-1 (Roche Applied Science, IN, USA) was used to analyze cell viability. Cells were seeded at 8000 cells per well in 96-well plates. Cell viability of the parental ovarian cancer cell lines with PAX2 expression and PAX2 knockdown ovarian cancer cell lines at 1, 2, 4, 6, 7, 8 days were measured by adding 10 μL of WST-1 reagent to each wells. The plates were incubated from 0.5 to 4 h in a humidified atmosphere (37 °C, 5% CO_2_). Plates were thoroughly shaken for 1 min, and then their light absorbance at 450 nm was measured against background controls using a microtiter plate reader.

### 4.5. Wound Healing Assay

Parental ovarian cancer cells (RMUGL, TOV21G) and PAX2 stable knockdown ovarian cancer cells were cultured to confluence or near confluence (>90%) in a 6-well dish. Cells were subsequently rinsed with phosphate-buffered saline and starved overnight in low serum medium (1.5 mL; 0.5%–0.1% serum in Dulbecco’s modified Eagle’s medium). On the day of the assay, a sterile 200 μL pipette tip was used to scratch a cross-shaped wound through the cell lawn. Cells were rinsed with phosphate-buffered saline, and the low serum medium was replaced with 1.5 mL of medium containing 10% fetal bovine serum. After the wounds were created, the cultures were photographed using phase contrast at 10× magnification at 0, 5, 10 and 24 h. The TScratch program (Computational Science and Engineering Laboratory, Zurich, Switzerland) [26] was used to measure the open areas and analyze the data.

### 4.6. Taqman Real-Time RT-PCR

One microgram of total RNA from each sample was used for first-strand cDNA synthesis using a high-capacity cDNA reverse transcription kit (Applied Biosystems, Carlsbad, CA, USA). Real-time PCR was performed on the synthesized cDNA using *G0S2*, *WFDC1*, *GREM1*, and *PAX2* Taqman gene expression assay mixes on the Bio-Rad CFX96 system (Bio-Rad Laboratories, Hercules, CA, USA). All results were normalized using cyclophilin A.

### 4.7. Western Blot Analysis

Cytoplasmic and nuclear proteins were extracted as described previously [45]. Western blots were performed with rabbit polyclonal anti-*PAX2* antibody (Zymed Laboratories, San Francisco, CA, USA) at a 1:500 dilution; rabbit polyclonal anti-Annexin AI antibody 71-3400 (invitrogen, Camarillo, CA, USA) at a 1:1000 dilution; rabbit polyclonal PARP-1/2 (H-250) antibody sc-7150 (Santa Cruz Biotechnology, Santa Cruz, CA, USA) at a 1:10,000 dilution; mouse monoclonal anti-beta-actin antibody (Sigma-aldrich Inc., St. Louis, MO, USA) at a 1:10,000 dilution; goat anti-mouse IgG-horseradish peroxidase sc-2005 (Santa Cruz Biotechnology) at a 1:10,00 dilution; and goat anti-rabbit IgG-horseradish peroxidase sc-2004 (Santa Cruz Biotechnology) at a 1:10,000 dilution. The bound antibodies were detected using an Amersham ECL Western blot detection reagent kit (GE Healthcare, Fairfield, CT, USA). Nuclear expression of PAX2 was normalized with nuclear expression PARP-1/2. Total protein expression of Annexin AI was normalized with beta-actin expression.

### 4.8. Reverse Phase Protein Array (RPPA)

Cell lysates were extracted by using lysis buffer and were serially diluted four times from undiluted to 1:16 dilution before they were arrayed on nitrocellulose-coated slides in an 11 × 11 format. Samples were probed with antibodies by a catalyzed signal amplification system and visualized by a diaminobenzadine colorimetric reaction. Slides were scanned on a flatbed scanner to produce a 16-bit tiff image. Spots from the tiff images were identified, and their density was quantified using MicroVigene (VigeneTech Inc., Carlisle, MA, USA). Relative protein levels for each sample were determined by interpolation of each dilution curve from the “standard curve” (supercurve) of the slide (antibody). All the data points were normalized for protein loading and transformed to linear values designated as “linear after normalization”. The “linear after normalization” values were then transformed to natural log values and median-centered for hierarchical cluster analysis. Samples were probed with 217 antibodies. Based on our QC samples which were defined by the software, only 207 antibodies were included in the data analysis. A heat map was used to express overall patterns.

### 4.9. Allophycocyanin-Annexin V Staining

One million cells were aliquoted into centrifuge tubes. Cells were centrifuged, and the supernatant was decanted. One hundred microliters of diluted (1:20 dilution) Annexin V (BD Pharmingen, Bedford, MA, USA) were added to each sample, followed by incubation at room temperature in the dark for 15 min. Precipitates were washed with the Annexin V binding buffer and resuspended in 400 μL binding buffer. Annexin V expression was determined using a FACSCalibur flow cytometer (Becton Dickinson, Mountain View, CA, USA), and single color samples were used to set compensation on the flow cytometer. Data were analyzed using the Becton Dickinson CellQuest Pro software package.

### 4.10. Transfection of Ovarian Cancer Cell Lines with PAX2 Full-Length cDNA Clone

Full-length PAX2 cDNA (pCMW-Myc-PAX2) clone and vector (pCMV-Neo) were purchased from Origene (Rockville, MD, USA). Ten thousands cells in each well of a 96 well plate were transfected with 0.1 μg DNA using Lipofectamin 2000 reagent (Life Technologies, Grand Island, NY, USA). After 3 days, cell survival was measured with WST-1 assay.

### 4.11. Immunostaining of PAX2 in Non-Serous Ovarian Carcinomas Paraffin Sections

Immunohistochemistry of paraffin embedded tissue was conducted to determine PAX2 protein expression in patient samples. Paraffin-embedded specimens were sliced into 5-μm sections and the histologic subtypes were confirmed by a pathologist (MD) with specialty in gynecologic malignancies. Following deparaffinization and rehydration, antigen retrieval was performed using citrate buffer in a decloaking chamber at the following settings: 121 °C for 3 min and 95 °C for 1 min. (Biocare Medical, Concord, CA, USA). Staining of the slides was conducted using the Lab Vision Autostainer 360 (Thermo Scientific, Waltham, CA, USA). A PAX2 rabbit polyclonal antibody (Invitrogen, Camarillo, CA, USA) was used along with the Envision + System-HRP Labelled Polymer Anti-Rabbit (Dako, Carpinteria, CA, USA). Slides were also counterstained with hematoxylin.

## 5. Conclusions

In this study, we demonstrated that knockdown PAX2 expression in ovarian cancer cells with high level of PAX2 expression is associated with reduced cell proliferation and motility. However, over-expressing PAX2 in PAX2-negative ovarian cancer cells suppressed their growth. In summary, PAX2 could have both oncogenic or tumor suppression functions, which will depend on the genetic content of ovarian cancer cells. Further investigation is warranted.

## Supplementary Files

Supplementary File 1 Supplementary Information (DOC, 229 KB)

Supplementary File 2 Table S1. Differentially expressed genes between PAX2 knockdown (XLSX, 18 KB)
