# Supplementary material for: PAX2 Expression in Ovarian Cancer
Source: Int J Mol Sci. 2013 Mar 15;14(3):6090–105. doi: 10.3390/ijms14036090 (PMC3634442; doi:10.3390/ijms14036090)
Supplement: Supplementary File 1 — Supplementary Information (DOC, 229 KB) [file ijms-14-06090-s001.doc]

Supplementary Information

**Figure S1.** Relative expression of *PAX2* in ovarian carcinomas with different histological types.

© 2013 by the authors; licensee MDPI, Basel, Switzerland. This article is an open access article distributed under the terms and conditions of the Creative Commons Attribution license (http://creativecommons.org/licenses/by/3.0/).
